# Selection and introgression facilitated the adaptation of Chinese native endangered cattle in extreme environments

**DOI:** 10.1111/eva.13168

**Published:** 2020-12-14

**Authors:** Xinfeng Liu, Zhaohong Li, Yubin Yan, Ye Li, Hui Wu, Jie Pei, Ping Yan, Ruolin Yang, Xian Guo, Xianyong Lan

**Affiliations:** ^1^ College of Animal Science and Technology Northwest A&F University Yangling China; ^2^ College of Life Sciences Northwest A&F University Yangling China; ^3^ Lanzhou Institute of Husbandry and Pharmaceutical Sciences Chinese Academy of Agricultural Sciences Lanzhou China

**Keywords:** adaptation, cattle, endangered, extreme environment, introgression, SNPs

## Abstract

Although persistent efforts have identified and characterized a few candidate genes and related biological processes with potential functions in the adaptation of many species to extreme environments, few works have been conducted to determine the genomic basis of adaptation in endangered livestock breeds that have been living in extreme conditions for more than thousands of years. To fill this gap, we sequenced the whole genomes of nine individuals from three Chinese native endangered cattle breeds that are living in high‐altitude or arid environments. Phylogenetic and evolutionary history analyses of these three and other six breeds showed that the genetic structure of the cattle populations is primarily related to geographic location. Interestingly, we identified pervasive introgression from the yak to Zhangmu cattle (ZMC) that cover several genes (e.g., *NOS2*, *EGLN1* and *EPAS1*) involved in the hypoxia response and previously identified as positive selection genes in other species, which suggested that the adaptive introgression from yak may have contributed to the adaptation of ZMC to high‐altitude environments. In addition, by contrasting the breeds in opposite living conditions, we revealed a set of candidate genes with various functions from hypoxia response, water metabolism, immune response and body shape change to embryo development and skeletal system development, etc., that may be related to high‐altitude or arid adaptation. Our research provides new insights into the recovery and adaptation of endangered native cattle and other species in extreme environments and valuable resources for future research on animal husbandry to cope with climate change.

## 1 INTRODUCTION

Cattle have been regarded as one of the most important animals domesticated by humans since the Neolithic period because they provide meat, milk, leather and traction for farming and transportation (Zhang et al., 2013). Based on genetic characteristics and phenotypic differences, most modern cattle breeds are divided into two groups, humpless taurine (*Bos taurus*) and humped indicine (*Bos indicus*) cattle, which were domesticated in the Fertile Crescent and the Indus Valley, respectively (Bickhart et al., 2016; Hiendleder et al., 2008). There are 53 native cattle breeds in China, which are further classified into three groups by their geographical locations, morphologies and sex chromosome polymorphisms: (a) southern cattle breeds largely descend from the indicine lineage, (b) northern cattle breeds belong to the taurine lineage and (c) cattle breeds from central China originated from taurine × indicine cattle hybrids (Lei et al., 2006; Zhang, 2011). The diverse geographic conditions and climates in China have resulted in some of the domestic breeds of cattle adapting to different extreme environments during evolution. For example, Zhangmu cattle (ZMC) has long been distributed in Nyalam County, Gyirong County and Tingri County of Tibet, China, and is one of a few domestic cattle breeds living at an altitude of about 5,000 m in the world (Zhang, 2011). ZMC was suspected to be a hybrid breed formed from ancient hybridization of taurine cattle, indicine cattle and yak in natural conditions (Zhang, 2011). Anxi cattle (AXC), named after the former Anxi County in Gansu Province, is a local breed endemic to the Gobi Desert of the “ancient Silk Road‐Hexi Corridor.” Due to the extremely harsh environment, AXC is thought to be the only local cattle breed living in this area (Zhang, 2011). Qaidam cattle (QDMC), another local taurine breed, which was domesticated from ancient Mongolian cattle with a strong resistance to alpine climates and mosquitos, is mainly distributed on the edge of Qaidam Basin (approximately 3,000 m in altitude) in Qinghai Province. QDMC has adapted to swamps and becomes a valuable genetic resource in this region (Zhang, 2011). However, these breeds are threatened by rapid climate changes, such as global warming in recent years (Easterling et al., 2000). In addition, increased human activities are another factor contributing to the decline in the species (Chen et al., 2019). It was estimated that the numbers of ZMC and AXC are less than 300 and thus on the verge of extinction. The number of QDMCs is less than 9,000 and is endangered (Zhang, 2011).

In recent years, genome‐wide selection analysis has been performed to identify adaptive genetic variations in a variety of domesticated animals living in extreme environments, resulting in a lot of important findings. Tibetan cattle has received the yak alleles of *EGLN1*, *EGLN2* and *HIF3α* in the HIF pathway by introgression (Wu et al., 2018). The genes involved in mitochondrial oxidative phosphorylation and hypoxic response have played a crucial role in the adaptation of yak to the extreme environment of the plateau (Qiu et al., 2012). *SOCS2* and *GPX3* were shown under strong positive selection in Tibetan sheep and Taklimakan desert sheep, respectively (Yang et al., 2016). More recently, a systematic investigation on the genetic mechanism underlying the adaptation of domestic animals (including Tibetan mastiff, Tibetan pig, Tibetan sheep, Tibetan goat, Tibetan horse, Tibetan cattle) to the Qinghai Tibet Plateau revealed that the convergent evolution of some genes, such as *EPAS1*, might have played an important role for human and many domestic animals adapting to plateau environment (Wu et al., 2018). These studies have led to a better understanding of the genetic mechanisms underlying species adaptation to extreme environments. However, to our knowledge, little research has characterized the genetic adaptations of endangered cattle to extreme environments based on whole‐genome deep sequencing.

In this study, for the first time, we used deep whole‐genome resequencing data to explore adaptive genetic mechanisms in three endangered cattle breeds living in either extremely high‐altitude or arid environments. These studies are of great significance for the conservation and restoration of endangered livestock genetic resources.

## 2 MATERIALS AND METHODS

### 2.1 Ethics statement

According to “Guidelines on Ethical Treatment of Experimental Animals (2006) No. 398,” the sampling procedures were in compliance with the Ministry of Science and Technology, China. All procedures conducted in this study were approved by the Northwest A&F University Experimental Animal Manage Committee.

### 2.2 Sample collection and sequencing

A total of nine samples from Zhangmu cattle (ZMC, *n* = 3), Qaidam cattle (QDMC, *n* = 3) and Anxi cattle (AXC, *n* = 3) were collected (Figure S1a; Table S1). The ear tissue of each individual was used to extract DNA. Using the Illumina standard protocol, genomic libraries with insert sizes of ~350 bp were constructed and sequenced by the Illumina HiSeq System (Illumina). In addition, the genome sequence data of 24 individuals were downloaded from the NCBI database, including Tibetan cattle (TC, *n* = 5), Kazakh cattle (KZKC, *n* = 3), Mongolian cattle (MGC, *n* = 3), Wenling cattle (WLC, *n* = 2), Wannan cattle (WNC, *n* = 2), Leiqiong cattle (LQC, *n* = 3) and six Yaks (Figure S1a; Table S1).

### 2.3 Reads mapping and variation identification

All cleaned pair‐end sequence reads were mapped against the reference bovine genome (UMD 3.1; Zimin et al., 2009) using Burrows‐Wheeler Aligner (BWA 0.7.5a) software (Li & Durbin, 2009) with the parameters “BWA aln ‐o 1 ‐L ‐i 5 ‐e 4 ‐n 2.” Aligned sequences were converted to raw BAM files using SAMtools for sorting and duplicating (Li & Durbin, 2009). Next, Genome Analysis Toolkit (GATK, version 3.7) UnifiedGenotyper (McKenna et al., 2010) was used to identify single nucleotide polymorphism (SNPs) with default settings. The raw SNPs were filtered by requiring a minimum coverage depth of 5 and a maximum of 100, a minimum RMS (root mean square) mapping quality score of 40 and no gap present within a 3‐bp window. Finally, we only retained high‐quality autosomal bi‐allelic SNPs for subsequent analysis. SNPs variants were annotated using ANNOVAR (Wang et al., 2010).

### 2.4 Linkage disequilibrium analysis

The values of identity by state (IBS) for all the samples were evaluated using PLINK v1.90 (Purcell et al., 2007). Genome‐wide linkage disequilibrium (LD) of cattle was calculated as the mean‐squared correlation coefficient (*r^2^*) values for pairwise markers using Haploview v4.2 software with the parameters “‐maxdistance 200 ‐minGeno 0.5 ‐dprime ‐missingCutoff 1 ‐minGeno 0.6 ‐minMAF 0.05 ‐hwcutoff 0.001 ‐dprime” (Barrett et al., 2005).

### 2.5 Population differentiation and genetic structure analysis

The phylogenetic relationship of cattle was inferred based on the high‐quality autosomal bi‐allelic SNPs using the neighbour‐joining (NJ) method in PHYLIP (v3.695; http://evolution.genetics.washington.edu/phylip.html). In this analysis, *p*‐distance was used and the produced phylogeny was visualized using FigTree v1.4.4 (http://tree.bio.ed.ac.uk/software/figtree/). The smartPCA program in EIGENSOFT (v7.2.10; Patterson et al., 2006) was used for principal component analysis (PCA) of the 27 individuals. ADMIXTURE was used to infer the population structure (Alexander et al., 2009). The cluster number *K* was set to 2 or 3, with 200 iterations for each run. The degree of genetic differentiation between any two groups of breeds was measured by *F*_ST_ (Weir & Cockerham, 1984).

### 2.6 Inference of demographic history

Pairwise sequential Markovian coalescent (PSMC) model was used to infer the history of effective population size (*N*_e_) of the native cattle over the last one million years (Li & Durbin, 2011). The parameter was set to “‐N30 ‐t15 ‐r5 ‐p ‘4 + 25*2 + 4 + 6’.” In addition, the multiple sequential Markovian coalescent (MSMC) model (Schiffels & Durbin, 2014) was used to infer the divergence time between two breeds of interest (samples marked in Table S2). Before the MSMC analysis, all the individuals were phased using Beagle (version 3.3.2; Browning & Browning, 2011). We defined the estimated divergence time between a pair of breeds as the first time point at which the cross‐coalescence rate was at 0.5. Both MSMC and PSMC used the same parameters generation time (*g*: 6 years) and average mutation rate (*μ*: 1.26 × 10^−8^ per base per generation) as used in Chen et al. (2018).

### 2.7 Detection of admixture

To infer the migration events of yak and nine native cattle breeds, a population‐level phylogeny analysis was performed using TreeMix (v.1.12; Pickrell & Pritchard, 2012). The program inferred an ML tree for the nine native cattle breeds (27 individuals) and the yak as the out‐group, and then, the residual matrix was used to identify pairs of populations that showed poor fits in the ML tree. From 0 to 5, migration events were gradually added to the ML tree until 99% of the variance between the breeds could be explained. The command parameter was “‐bootstrap ‐k 10,000 ‐m migration events.” To further detect the evidence of admixture across populations, an ancestry graph and three‐population (*f3*) tests implemented in TreeMix were utilized to examine the presence of admixture (Pickrell & Pritchard, 2012; Reich et al., 2009).

### 2.8 Detection of pairwise identity by descent

To examine the genetic contribution from yak to ZMC, the genomic regions of the potential introgression between the yak and ZMC were detected by calculating the frequency of shared identical by descent (IBD) by referring to Bosse et al. (2014). Specifically, the autosomal sequences of all individuals were phased and IBD chunks were inferred using Beagle fastIBD (version 3.3.2) under default settings (Browning & Browning, 2011). Next, the IBD chunks between donator (yak, *n* = 6) and recipient (ZMC, *n* = 3) population or between ZMC and the remaining cattle population (*n* = 24) were extracted to count the frequency of shared IBD with a window size of 20 kb and a step size of 10 kb. The relative frequency of IBD (rIBD) was then calculated as follows: rIBD value (yak and ZMC) − rIBD value (ZMC and remaining cattle breeds). Ultimately, genomic regions with negative rIBD values were identified as potential introgression from yak to ZMC. We further inspected a few potential introgression regions of interest with regard to *F*_ST_, *θ_π_*, Tajima's *D* (Tajima, 1989), *Dxy* (Nei, 1987) and the topology of induced NJ trees.

### 2.9 Selective signals in extreme environments and nonextreme environments

For each of the nine cattle breeds, we first assigned it into two opposite groups (extreme environment groups vs. nonextreme environment groups) based on either the altitudes (altitude >3,000 m vs. <1,300 m) or the annual precipitation (average annual precipitation <350 mm vs. >500 mm) information (Table S13). Next, to detect the potential local selection regions of cattle genome corresponding to a certain extreme environment, we performed a sliding window analysis (with 50‐kb windows and 10‐kb steps) using three metrics: *θ_π_*, *F*_ST_ and Tajima's *D*. An idea region with strong positive section signal would be characteristic of high relative polymorphism level, high *F*_ST_ between groups and a lower negative Tajima's *D* in the extreme environment group than in the nonextreme environment group. The average values of *θ_π_*, *F*_ST_ and Tajima's *D* were calculated for each window using VCFtools (Danecek et al., 2011). It must be noted that apart from selection, demography can also influence Tajima's *D* value, so we should combine Tajima's *D* with other population genetic metrics (such as *θ_π_*) to infer whether a local region is likely to be a target of positive selection (Tajima, 1989). We considered the windows with the top 5% *F*_ST_ and relative polymorphism levels simultaneously as the candidate regions subjected to positive selection. Finally, DAVID 6.7 (http://david.abcc.ncifcrf.gov/) was used for GO and KEGG analyses of overlapping genes in the candidate regions.

## 3 RESULTS AND DISCUSSION

### 3.1 Whole‐genome genetic variation

We collected three endangered cattle breeds from Tibet and north‐west of China (Figure S1a; Table S1). Whole‐genome resequencing generated a total of 7.57 billion paired‐end reads with an insert size of approximately 350 bp on average. Alignment with the reference genome of *B. taurus* (UMD3.1) showed an average depth of 35.12**×** (Table S2). To place these cattle into a more detailed context, we analysed these genomic data with available resequencing data from 18 individuals representing six different breeds living in northern or southern China (Figure S1a; Table S1). We detected approximately 46 million SNPs among 27 individuals. After quality control, approximately 25 million high‐quality SNPs for subsequent analyse, 87.61% of which were present in the dbSNP database (Table S3), indicating the high reliability of the called SNPs in this study. At the chromosome level, the longer the chromosome, the more the SNPs (Figure S2a; Table S4). At the breed level, LQC has the most total SNPs, followed by ZMC, WLC, WNC, TC, MGC, AXC, QDMC and KZKC. Obviously, the number of private SNPs of ZMC is significantly higher than other northern cattle breeds, indicating that ZMC may have higher genetic diversity (Figure S2b; Table S3). Approximately 9.4 million SNPs are shared between the three groups (Figure S2c). The genomic distribution of the high‐quality SNPs indicated that a majority of them are located in intergenic regions but with only 0.68% in the exon regions (Table S5). For exonic SNPs, 64,679 SNPs and 104,080 SNPs were synonymous and nonsynonymous, respectively, which resulted in a nonsynonymous/synonymous ratio of 0.621 (Table S5).

### 3.2 Genomic variation and linkage disequilibrium

The genome‐wide nucleotide diversity (*θ_π_*) for the nine native cattle breeds was between 1.54** × **10^−3^ and 2.37** × **10^−3^, which was comparable with the values from other mammals and humans (Figure S2d; Tables S6 and S7). Remarkably, ZMC showed a higher nucleotide diversity than the other five taurine cattle breeds (Figure S2d; Table S6). The genetic diversity was also reflected in the number of heterozygous SNPs, that is, although the number of heterozygous SNPs in ZMC is lower than that in the three southern indicine cattle breeds, the heterozygosity of ZMC is the highest among the analysed northern taurine cattle breeds (Table S3). Inbred individuals were not observed in the investigated cattle samples according to the IBS score (IBS < 0.9; Table S8). LD analysis revealed that QDMC and AXC showed a slow decay rate and high level of LD, whereas the TC exhibited a rapid decay rate and a low level of LD (Figure S1b), which seems to be consistent with our finding that TC had the largest *N*_e_ among the cattle breeds to be analysed (see Section 3.4 for details).

### 3.3 Population genetic structure

**FIGURE 1:**
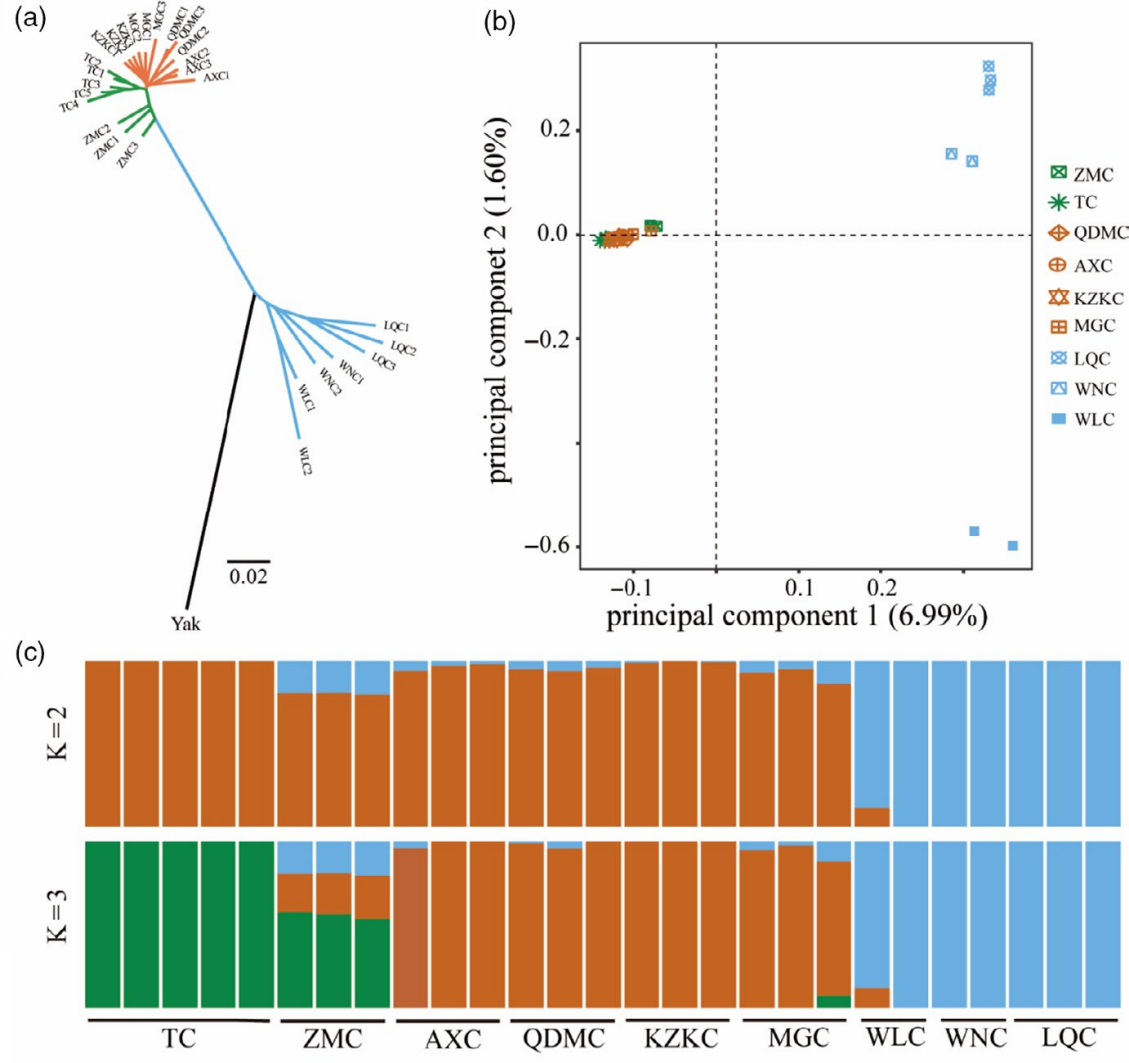
Population genetic analyses. (a) Neighbour‐joining (NJ) phylogenetic tree of nine native cattle breeds (27 individuals) based on *p*‐distances between individuals. The yak (*Bos grunniens*) was used as out‐group. (b) Principal component analysis clustering pattern of individuals. Principal components 1 and 2 were used to represent the relationships among the 27 individuals. (c) ADMIXTURE result showing the genetic structure of Chinese native cattle. The length of each colour fragment represents the proportion of the individual genome inferred from the ancestral population (*K* = 2, 3)

To determine the phylogenetic relationship among the nine Chinese native breeds containing a total of 27 cattle individuals, we built a neighbour‐joining (NJ) tree based on the whole‐genome SNPs in autosomes using yak (*Bos grunniens*) as an out‐group (Figure 1a). Notably, these native cattle samples were assigned to southern indicine cattle breeds (LQC, WNC and WLC) and northern taurine cattle breeds (TC, QDMC, MGC, KZKC and AXC) with a high level of the fixation index (*F*_ST_ = 0.260), consistent with their ancestry and geographical distribution. Interestingly, ZMC is located between the two clades of indicine cattle and taurine cattle, and is closer to taurine cattle clade, suggesting that the ZMC has ancestor components of taurine and indicine (Figure 1a). The indicine cattle and taurine cattle were clearly separated by PCA (Figure 1b). We further performed ADMIXTURE analysis to explore the genetic composition of the samples with the number of clusters (*K*) varying from 2 to 3 (Figure 1c). When *K* = 2, the samples were clearly divided into two groups, reflecting the divergence of taurine and indicine cattle, congruent with their geographic distribution and ancestral sources (Figure 1c). At *K* = 3, ZMC showed a clear admixture signature with genome ancestry with taurine and indicine cattle genetic background (Figure 1c). These results are largely consistent with the findings from the NJ tree and PCA and again suggested that ZMC possessed a unique evolutionary history.

### 3.4 Inference of demographic history

**FIGURE 2:**
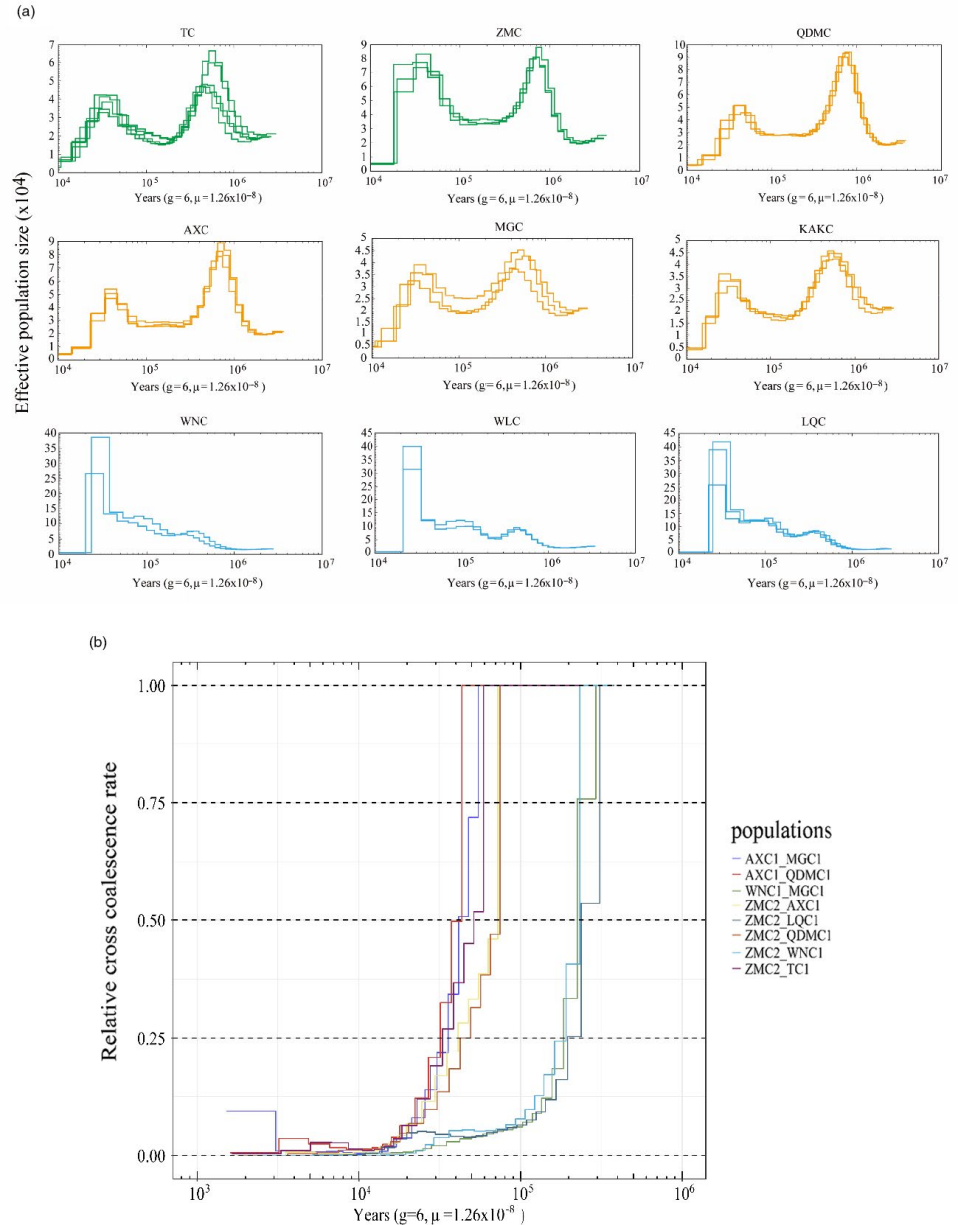
Demographic history of the native cattle. (a) History of effective population size of nine cattle breeds inferred from pairwise sequential Markov coalescence (PSMC). The PSMC model was used to infer the effective population size of the nine native cattle breeds over the past 10^6^ years, the generation time (*g* = 6) and the mutation rate (*μ* = 1.26 × 10^−8^), with one line per individual. The *x*‐axis moves to the right to show the population size further back in time. (b) The relative cross‐coalescence rates between the breeds were estimated using multiple sequential Markovian coalescent (MSMC), with four haplotypes per pair, generation time (*g* = 6) and mutation rate (*μ* = 1.26 × 10^−8^)

The population history for the 27 individuals was predicted using the PSMC model. In contrast, with the ancestral *N*_e_ of southern cattle breeds that might have mainly experienced a distinct decline during evolution, the North cattle breeds seemed to have undergone two distinct declines (Figure 2a and Figure S3). The first declines occurred ~0.9 Mya, coinciding with the Xixiabangma Glaciation (XG, 1.1–0.8 Mya; Zheng et al., 2002). After a long decline, the population of these taurine cattle breeds gradually recovered and ultimately peaked at ~40,000 years ago. Noticeably, although ZMC, such as other breeds of North ancestry, had a smaller *N*_e_ than that of the southern breeds, its *N*_e_ was significantly higher than the remainder of the northern taurine cattle during this peak period (Figure 2a and Figure S3). We speculate that this may be due to the mixture events between the ancestral ZMC and northern taurine cattle and southern indicine cattle, as reflected from the clustering pattern of the STRUCTURE analysis, resulting in a higher genetic diversity of ZMC than of other northern taurine cattle. Subsequently, the population size of all the breeds gradually decreased to a small number during the LGM (~20,000 years ago; Figure 2a and Figure S3; Lorenzen et al., 2011). Geological research shows that it was at the maximum value of the glacial ~20,000 years ago, and this extremely cold climate made it difficult for animals to survive and the *N*_e_ of animals declined sharply (Groenen et al., 2012). In addition, studies have also shown that during this period, human beings have expanded to Eurasia and America, occupying the living space of animals, resulting in the decline of the number of animals (Nielsen et al., 2017). We noticed that this period happens to coincide with the domestication of cattle. It was proposed that the domestication of an animal from their wild ancestor can cause an ancient bottleneck of these species (Wang et al., 2014). Therefore, we speculate that the domestication in itself may be another factor for the decline in the effective population size of livestocks. Overall, consistent with the previous studies, our analysis provided an additional line of evidence for the changed effective population size in taurine cattle (Chen et al., 2018; Mei et al., 2017).

We used MSMC model to infer the divergence times between the three endangered breeds and other breeds inspected in this study (Figure 2b; Schiffels & Durbin, 2014). As the relative cross‐coalescence rate (0.5) showed, ZMC may have diverged from the indicine (LQC and WNC) cattle ~0.23 Mya during the Penultimate Glaciation (0.30–0.13 Mya; Figure 2b; Zheng et al., 2002). This inferred divergence time overlapped with a divergence time of taurine and indicine lineages (332–117 kya) estimated from the molecular genetic data (Figure 2b; Achilli et al., 2008; Bradley et al., 1996). Next, we found that the divergence time between ZMC and other northern cattle breeds was ~70–30 kya, which was obviously later than the divergence time between ZMC and southern cattle breeds (Figure 2b). The analysis results of PSMC and MSMC further supported the results of population genetic structure analysis.

### 3.5 Inference of genetic admixture

The above analysis suggested that the increased genetic diversity in ZMC may have been due to an ancient gene flow between the ancestors of ZMC and the other cattle breeds. To further determine the signature of population admixture, TreeMix was used to evaluate possible admixture events among the nine local cattle breeds (Figure S4). An admixture graph obtained after adding five migration events optimally improved the model fit since it explained more than 99% of the genetic variance across populations (Figure S4). When *M* = 1–4, the obvious gene flow can be found from the northern taurine cattle to the ZMC (Figure S4). However, when *M* = 5, the method instead inferred weak gene flow from the ancestors of the southern indicine cattle to the ancestors of ZMC (Figure S4). Consequently, the TreeMix analysis suggested that the genome of extant ZMC might involve a mix of the genomic background of both indicine cattle and taurine cattle. To further evaluate the presence of admixture, we computed *f3* statistics on all possible population triples (Population A; Population B; and Population C) using the TreeMix package. The extreme negative *f3* statistic values indicate gene flow to Population A from both populations B and C. For MGC, this analysis produced six extreme negative *Z*‐scores (−55.72, −49.76, −45.70, −38.62, −31.30 and −30.89) when using the populations of KZKC|LQC, WNC|KZKC, WLC|KZKC, TC|LQC, WNC|TC and WLC|TC as sources, respectively, which seemly suggested MGC with a complex admixture history (Table S9), while we cannot rule out the possibility that the gene flow is from only one or two source breeds to MGC genome. In addition, using WNC and KZKC as sources produced *Z*‐scores of −33.49 for ZMC as an admixture population (Table S9), providing additional evidence of extensive gene flow during the evolution history of ZMC that contributed to the rich genetic diversity of this breed.

### 3.6 Introgression mapping between yak and ZMC

**FIGURE 3:**
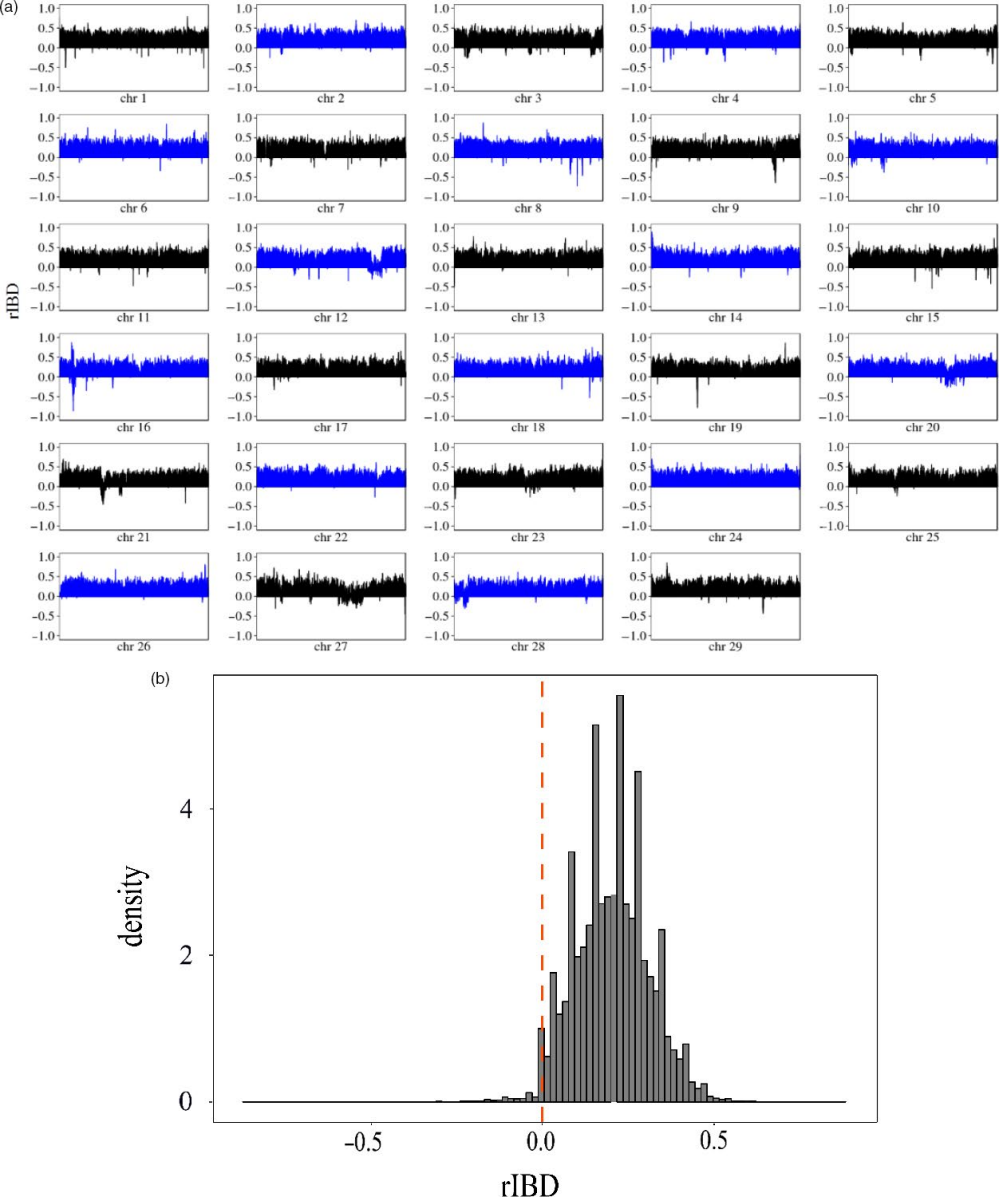
Genome‐wide pattern of introgression between the Zhangmu cattle (ZMC) and yak. (a) The *x*‐axis represents the whole chromosome, and the *y*‐axis represents the rIBD value. Negative rIBD indicated that the signal of introgression came from the yak. (b) Distribution of rIBD in ZMC and the yak (−1 to 0) or control population (0 to 1)

**FIGURE 4:**
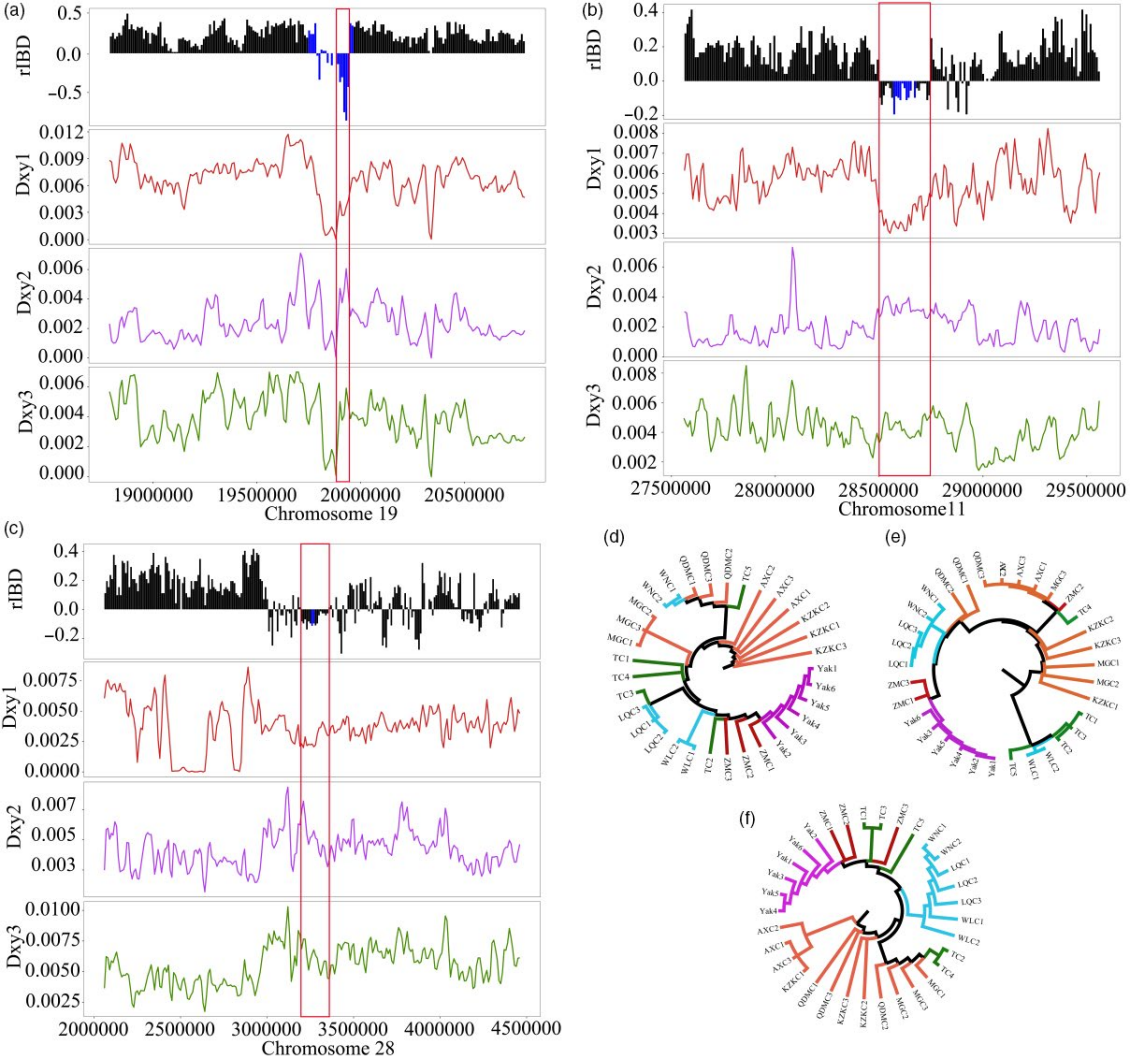
*Dxy* and phylogeny analysis for adaptive introgression regions. (a–c) rIBD, *Dxy1* (between Zhangmu cattle [ZMC] and yak), *Dxy2* (between ZMC and taurine cattle) and *Dxy3* (between ZMC and indicine cattle) values of the three continuous introgression regions in the chromosome 19 (19860000–19960000), chromosome 11 (28500000–28760000) and chromosome 28 (3200000–33700000), respectively. (d–f) Phylogenetic tree for the continuous introgression regions, respectively. The continuous introgression regions of the chromosome 19, chromosome 11 and chromosome 28 are marked by red boxes and the loci of *EPAS1*, *NOS2* and *EGLN1* are marked by blue bars

**FIGURE 5:**
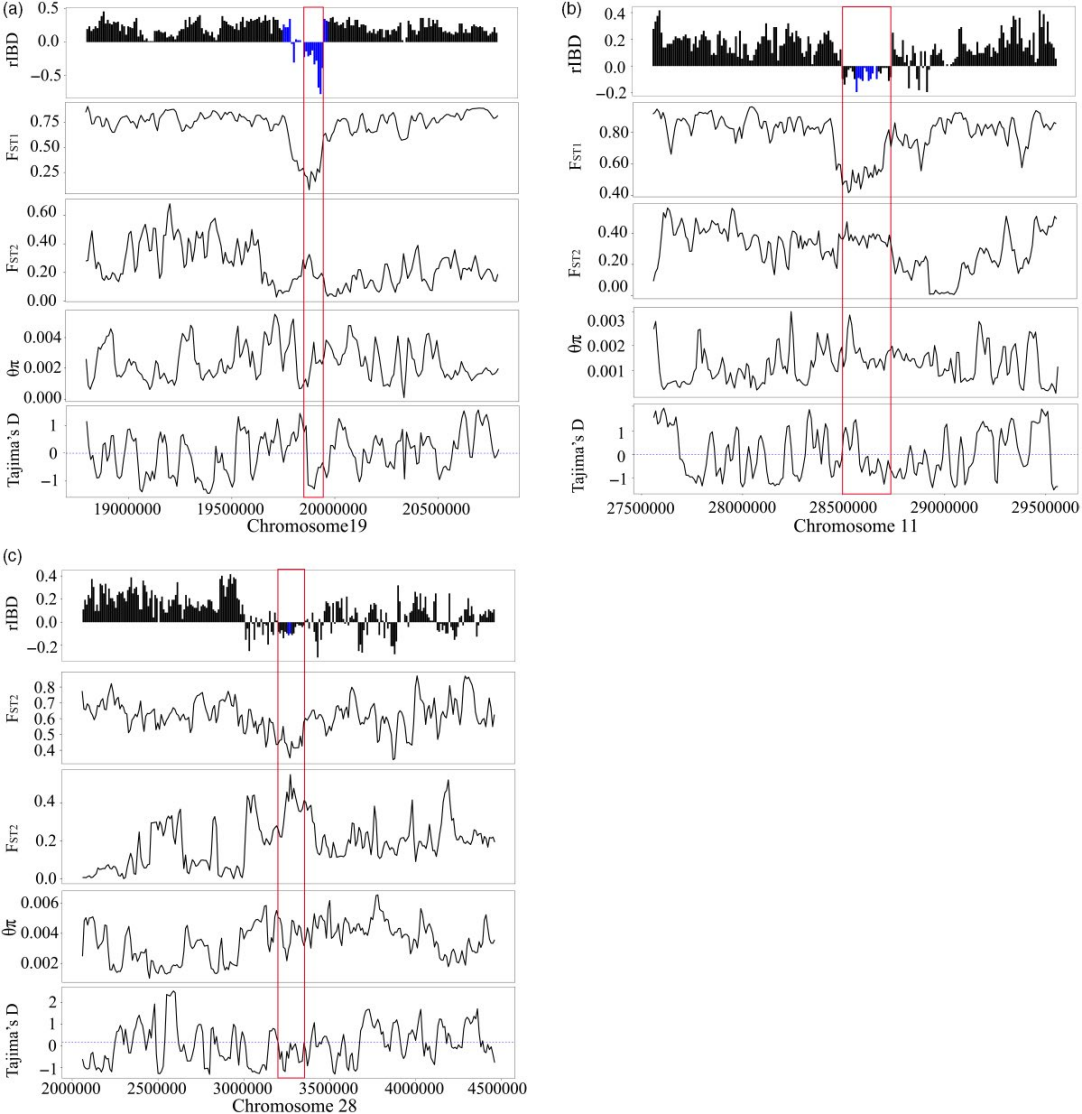
Adaptive introgression signals from yak to Zhangmu cattle (ZMC). (a–c) Distribution patterns of rIBD, *θ_π_* (ZMC), Tajima's *D*, *F*_ST1_ (between ZMC and yak) and *F*_ST2_ (between ZMC and indicine cattle) around the continuous introgression regions in the chromosome 19 (19860000–19960000), chromosome 11 (28500000–28760000) and chromosome 28 (3200000–33700000), respectively. The red boxes represent the continuous introgression regions, and blue bars represent the loci of *NOS2*, *EPAS1* and *EGLN1*

Considering that both yak and ZMC are endemic to the Qinghai–Tibet Plateau, we examined possible yak introgression into ZMC. To this end, we calculated the rIBD statistics to infer the genomic regions of ZMC that might have been introgressed from yak. The results showed that there were ~48 Mb (~1.8% of cattle genome) introgression regions (rIBD < 0) between ZMC and yaks, covering 380 protein‐coding genes (Figure 3a,b; Table S10). Noticeably, chromosome 3, chromosome 27 and chromosome 28 of ZMC contain several large introgression regions (Figure 3a and Figure S5), displaying a lower *F*_ST_ with yak than that in their adjacent nonintrogression regions (Figure S5). To consider the introgression process in the context of adaptive evolution, we performed enrichment analysis on the 380 genes overlapping with the introgression regions (Table S10). Accordingly, “olfactory transduction,” “hematopoietic cell lineage,” and “viral myocarditis” KEGG pathways, and “antigen processing and presentation,” “MHC protein complex,” “MHC class I protein complex” and “immune response” GO terms are the most significant pathways or GO terms with overrepresented candidate genes (Table S11). Interestingly, *NOS2*, *EPAS1* and *EGLN1* are annotated to “response to hypoxia” GO term (Table S11). *NOS2*, ~197 kb in length, is overlapped with a continuous introgression region (Chr19:19860000–19960000) showing the lowest rIBD value (Figure 4a). Phylogeny analysis and *Dxy* assessment further corroborated this continuous region was likely to be introgressed from yak to ZMC (Figure 4a,d; Table S12). Moreover, compared with the adjacent nonintrogression regions, this continuous introgression region tended to exhibit a lower level of Tajima's *D* in ZMC population, a smaller *F*_ST1_ between ZMC and yak, but a larger *F*_ST2_ between ZMC and indicine cattle breeds (Figure 5a). Meanwhile, although the *θ_π_* displayed a larger fluctuation across windows probably due to the very limited samples, it seems that the nucleotide diversity in this introgression region was lower than that in the immediately flanking nonintrogression regions (Figure 5a). Together, this pattern probably suggested an episode of ancient adaptive introgression. *NOS2* gene, encoding inducible nitric oxide synthase (iNOS), is responsible for the production of nitric oxide (NO) in organisms (Moncada & Higgs, 1991). Previous studies have shown that under hypoxic conditions, the expression of *NOS2* can be induced in many mammals and fish (Cameron et al., 2013; Kido et al., 2005; Tekin et al., 2010; Thompson et al., 2009; Yang et al., 2018). In addition, *NOS2* exhibits the strongest selection signal in the human high‐altitude Andean population and is significantly associated with cardiovascular development and function (Crawford et al., 2017). Moreover, the *NOS2* gene was detected in Ladakhi cattle play a pivotal role in association with high‐altitude adaptation through comparative transcriptome analysis (Verma et al., 2018). Incorporating these lines of evidence from our and previous studies, we cautiously speculate that the *NOS2* allele introgressed from yak may have driven the phenotypic evolution of ZMC.

Additionally, *EPAS1* and *EGLN1*, located within continuous 0.26 and 0.17 Mb introgression regions in chromosome 11 and chromosome 28, respectively, attracted our attention given their functions (Figure 4b,c). Both phylogenetic tree and *Dxy* analyses supported the two possible introgression events between yak and ZMC (Figure 4b,c,e,f; Table S12). *EPAS1* and *EGLN1* encode the hypoxia‐inducible factor HIF‐2 and prolyl 4‐hydroxylase 2 (PHD2), respectively, both of which are located at the core of the hypoxia‐inducible factor pathway and identified as key genes for hypoxia adaptation in Tibetan (Bigham & Lee, 2014; Lorenzo et al., 2014). Certain variants of *EGLN1* and *EPAS1* are associated with lower haemoglobin concentrations, which are beneficial for animals to avoid suffering from polycythemia (Xu et al., 2011). Obviously, the two introgression regions indeed showed a smaller Tajima's *D*, a lower level of differentiation between ZMC and yak but a higher level of differentiation between ZMC and indicine cattle breeds than there corresponding adjacent nonintrogression regions (Figure 5b,c). Again, the averaged *θ_π_* of the introgression regions seems to be lower, at least slightly, than that of their corresponding nonretrogression regions (Figure 5b,c), providing evidence for the positive selection of the two introgression regions. The yak has been living on the Qinghai–Tibet Plateau for millions of years and has evolved unique traits, such as the enlargement of the lungs and heart, to adapt to the extreme high‐altitude environment (Chen et al., 2014). Domestic cattle were introduced onto the Qinghai–Tibet Plateau through human migration only thousands of years ago. The domestic cattle may be challenged by the high‐altitude environment once they arrived at the Qinghai–Tibet Plateau. Luckily, the ancestors of domestic cattle obtained some foreign copies of homologous genes from yak; these genes, such as *EGLN1*, *EGLN2* and *HIF3a* from HIF pathways, have so important functions in the response to hypoxia that they may at least partially substitute the original alleles of specific cattle breeds, and eventually substantially contribute to their adaption in the new habitat (Chen et al., 2018; Wu et al., 2018). Possibly in a similar way, the *EPAS1* has been found to be introgressed from Denisovans into Tibetans and from Tibetan wolves into the Tibetan mastiff, leading to the adaptive evolution of corresponding species (Huerta‐Sánchez et al., 2014; Miao et al., 2017). Thus far, a few genes appear to be frequently adopted by different species to conquer extreme habitats. Remarkably, introgression may provide an opportunity for those genes as a whole into the genomes of corresponding species, eventually contributing to the adaptive evolution of various species independently. In sum, our results suggested that the adaptive introgression from other species (e.g., yak) may have influenced the phenotypic evolution of domestic cattle.

It should be noted that we relied on phased genomic data to perform the introgression mapping analysis. Given the limited samples of ZMC, it would be hard to identify the rare and low‐frequency haplotypes. Therefore, in this study, the detection of IBD that is based on the sharing of rare haplotype may be less accurate than it would be if using many more samples. To minimize this kind of risk that may lead to inflated results and spurious conclusions, we only retained two analyses whose input data are in the form of phased data, and all the other analyses were based on nonphased genomic data. Although the introgression analysis might not be completely confidential, these results at least provide a valuable resource of candidate genes for further experimental validation in the future, while we think that more samples are needed to draw a more reliable conclusion regarding the evolutionary history and selective forces of putative introgression of interest.

### 3.7 Adaptive mechanisms in high‐altitude environments

A more interesting question is to what extent the evolution of domestic cattle might be driven by selective forces related to environmental stress. To address this issue, we performed genome‐wide selection scan analysis using two contrasting groups of cattle breeds that have lived in either high‐altitude or low‐altitude environments (Table S13). Using the top 5% of the *F*_ST_ values and *θ_π_* ratios as cut‐offs, we obtained 1,056 candidate genes that might be related to high‐altitude stress to varying degrees (Figure S6a; Table S14). By referencing the GO and KEGG pathway annotations, we found six genes in the classical HIF‐1 (hypoxia‐induced factors) pathway and 11 genes belonging to the GO term “the cellular‐response/response to hypoxia” (Table S16), suggesting the potential roles of these genes to the hypoxia adaptation of high‐altitude cattle breeds. Among the candidate genes, *PTPN9* attracted our attention, that is it showed higher *F*_ST_, *θ_π_* ratio, more negative Tajima's *D* than its adjacent nonintrogression regions in the high‐altitude group, meaning a positive selection signature (Figure S6b). *PTPN9* is a member of protein tyrosine phosphatase (PTP) family with known functions in cell proliferation, differentiation and migration. PTPN9 exerts its role through dephosphorylating protein substances, such as NSF, FOXO1, ERBB2, EGFR and VEGFR2, promoting homotypic vesicle fusion (Huynh et al., 2004), mediating insulin signalling in hepatocytes (Cho et al., 2006), suppressing breast cancer cell growth (Yuan et al., 2010) and regulating endothelial cell function (Hao et al., 2012). Besides, *PTPN9* has been identified as a positively selected gene in Tibetan sheep, possibly exerting its function via an interaction with *EGFR* in the classic HIF‐1 pathway (Yang et al., 2016). Therefore, *PTPN9* is most likely to be an important gene for the adaptation of domestic livestock to extreme high‐altitude stress. In addition, seven genes in vascular smooth muscle contraction (VSMC) pathway and three genes in glycolysis/gluconeogenesis pathway (Table S16) were discovered. VSMC pathway functions in adjusting the diameter of blood vessels and the delivery of blood oxygen (Yang et al., 2016); glycolysis/gluconeogenesis pathway is particularly important for the energy metabolism of cattle under extreme hypoxic conditions. The recovery of these genes in this comparative analysis is consistent with a previous finding in the Tibetan antelope (Ge et al., 2013). The immune to response GO term also annotated some candidate genes, such as *IL6* gene (Table S16). *IL6* is an immune‐related regulatory gene, encoding interleukin‐6 cytokine. It can mediate the differentiation of macrophages in vivo, inhibit the production of inflammatory cytokines by macrophages and promote the survival and regeneration of damaged epithelium during inflammation (Grivennikov et al., 2009; Mauer et al., 2014). Therefore, the IL6‐mediated immune response seems to play an important role in disease resistance and adaptation to harsh conditions for high‐altitude cattle. Previous studies have revealed that genes involved in lipid metabolism play an important role in the adaptation of many high‐altitude organisms to cold environments (Chen et al., 2016; Cheviron et al., 2012; Qiu et al., 2015). In this respect, we found two lipid metabolism‐related genes, *B4GALNT1* and *PLIN2*, among the candidate genes (Table S16). Moreover, many genes with GO terms “embryo development” or “skeletal system development” were included in the candidate genes (Table S16). It has been reported that some genes with developmental functions undergo positive selection in other high‐altitude species, such as the Tibetan human population (Xi et al., 2016) and Tibetan sheep (Yang et al., 2016). Considering the observation that the cattle population on the Qinghai–Tibet Plateau has a smaller body size and low energy consumption rate than low‐altitude breeds, we speculate that the development regulatory network may be involved in the adaptive evolution of cattle at high‐altitude regions by regulating the physical form of cattle body. Overall, our results provide many candidate genes that might relate to the high‐altitude adaptation of cattle.

### 3.8 Adaptive mechanisms in arid environments

Next, we investigated the genomic selection signal that might be associated with the adaptation of cattle in arid environment by comparing the cattle breeds lived in arid and humid environments (Table S13). Using the same method, 871 candidate genes were screened (Figure S7a; Table S15). Among them, 286 genes are also among the 1,056 candidate genes screened using altitude information, suggesting that these genes might be involved in adaptive responses to diverse environmental stress. Among the 871 genes screened in arid stress, four, three and ten genes, respectively, are in the arachidonic acid (AA) metabolism pathway, the renin–angiotensin system pathway and the oxytocin signalling pathway (Table S17). Previous researches indicated that these signalling pathways play significant roles in regulating water retention and reabsorption in renal kidney cells and blood vessels (Breyer & Breyer, 2000; Maguire et al., 1997). Therefore, our analysis suggested that the arid adaptation of species may be achieved via a complex signalling network orchestrating downstream effector genes. To provide a concrete example, the gene *PLA2G4B*, a member in the AA metabolism pathway, displayed high *F*_ST_ and *θ_π_* ratio, and a lower Tajima's *D* value than the adjacent areas of this gene only for the arid group, indicating a signal of positive selection (Figure S7b). *PLA2G4B* belongs to the cytosolic phospholipase A2 protein family (Song et al., 1999). Previous studies have shown that genes located in the AA metabolism pathway play an important role in the conversion of AA to 19(S)‐HETE (an effective renal vasodilator that stimulates water reabsorption; Bradley et al., 1996; Jirimutu et al., 2012). Hence, we speculate that *PLA2G4B* might have experienced adaptive evolution since the ancestors of cattle breeds entered into extreme arid conditions. This finding is consistent with the observation that the other three genes in the AA metabolic pathway are under positive selection in Bactrian camels or sheep living in desert environments (Jirimutu et al., 2012; Yang et al., 2016).

In addition, six candidate genes belong to the pancreatic secretion pathway with the function of protein and carbohydrate digestion and absorption (Yang et al., 2016), which seems to well explain the characteristics of coarse feeding tolerance of livestock living in extremely arid conditions (Table S17). We also identified GO terms with diverse function annotations from immune response, lung development, skeletal system development, embryo development, response to xenobiotic stimulus and regulation of glucose metabolic process (Table S17), indicating the importance of body shape changes, energy optimization, disease defences and stress responses to survival in arid environments. The function of these GO terms is highly consistent with the small size and high saline‐alkali tolerance observed in sheep breeds in the Taklamakan Desert (Yang et al., 2016). Given the dry climate in north‐west China with high soil salinity levels and a lack of forage resources, it is reasonable to speculate that these pathways should have played important roles in adapting cattle to extreme drought stress. In general, given the persistence of global warming and the increasing incidence of drought, these candidate genes screened in this study would be a valuable resource for studying the adaptation of organisms to harsh environmental changes.

## 4 CONCLUSION

In this study, for the first time, we used deep genome‐wide polymorphism information on three native Chinese endangered cattle living in extreme environments (i.e., high‐altitude and arid areas in north‐west China) to conduct a comprehensive analysis of their genetic backgrounds. We determined the phylogenetic relationship of the native endangered cattle and identified a complex gene admixture history between multiple breeds in the Bos subfamily. In particular, we found that ZMC may have obtained foreign alleles of at least three core genes (*NOS2*, *ENGL1* and *EPAS1*) of the “response to hypoxia” biological process from the yak by introgression, which is of great significance for the adaptation of ZMC to extremely high‐altitude environments. Then, genome‐wide comparison analyses between native cattle breeds from extreme and nonextreme environments revealed a variety of genes with diverse functions that may jointly contribute to the adaptation of cattle in plateau and arid environments. Overall, this work revealed the evolutionary history and adaptive characteristics of endangered cattle breeds living in extreme environments, which will provide useful guidance for the development of reasonable conservation plans in the future.

## CONFLICT OF INTEREST

None declared.

## AUTHOR CONTRIBUTIONS

X. Lan and X. G. conceived the research. X. Lan, R. Y. and Z. L. designed the research. X. G. prepared animal samples. X. Liu and Z. L. collected and analysed the data with inputs from all authors. X. Liu drafted the manuscript. X. Lan., R. Y., X. Liu., Z. L and Y. Y. revised the manuscript. X. Lan and R. Y. supervised the project. All authors read and approved the final manuscript.

## Supporting information

Fig S1‐S7

Table S1‐S17

## Data Availability

Our whole‐genome Illumina sequencing reads have been deposited in the Sequence Read Archive (https://www.ncbi.nlm.nih.gov/sra) with the accession code (BioProject ID: PRJNA597241). The whole‐genome sequence data of native cattle and Yaks are available at National Center for Biotechnology Information using Sequence Read Archive numbers SRR5507243, SRR5507244, SRR5507245, SRR6024571, SRR6024572, SRR5507258, SRR5507259, SRR5507260, SRR5507261, SRR5507262, SRR5507263, SRR6234762, SRR6234763, SRR6024573, SRR6024574, SRR5507188, SRR5507189, SRR5507190, SRR2059950, SRR2059951, SRR2059952, SRR2059962, SRR2059963 and SRR2059964.
