# Supplementary material for: Selection and introgression facilitated the adaptation of Chinese native endangered cattle in extreme environments
Source: Evol Appl. 2020 Dec 14;14(3):860–73. doi: 10.1111/eva.13168 (PMC7980270; doi:10.1111/eva.13168)
Supplement: Supplementary file 1 — Fig S1‐S7 [file EVA-14-860-s002.pdf]

SUPPLEMENTARY MATERIAL

Supplementary figure

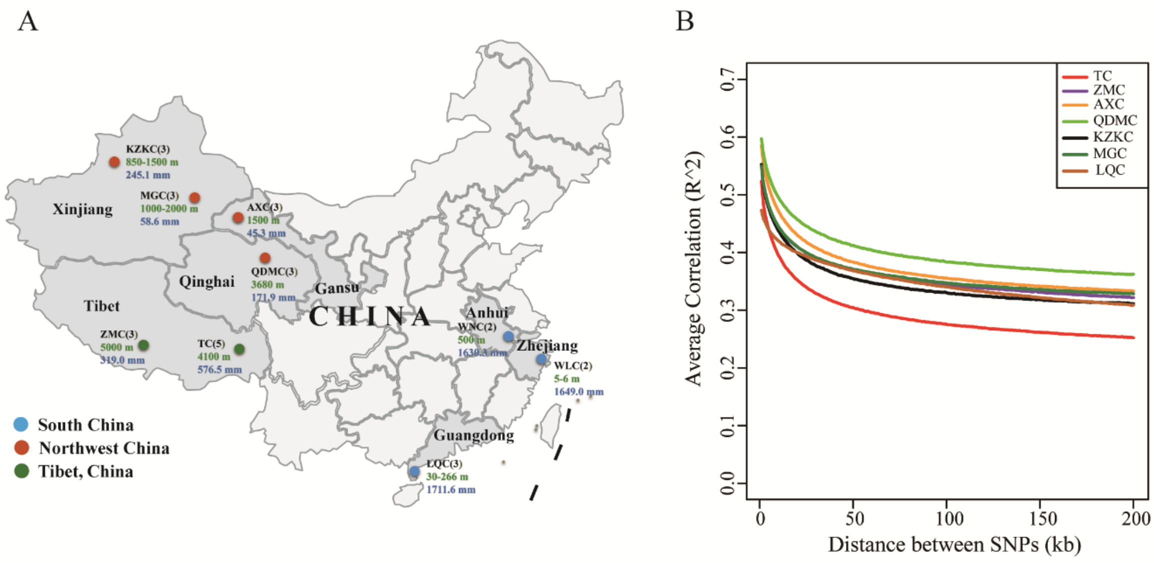

Figure S1. Geographic distribution and LD decay of native cattle breeds. (A) Geographical location of nine native cattle breeds, with green for elevation (m) and blue for precipitation (mm). (B) The linkage disequilibrium decay of 7 native cattle breeds was inferred, one line per breed. To minimize the influence of sample size, only breeds with at least three individuals were used in our study.

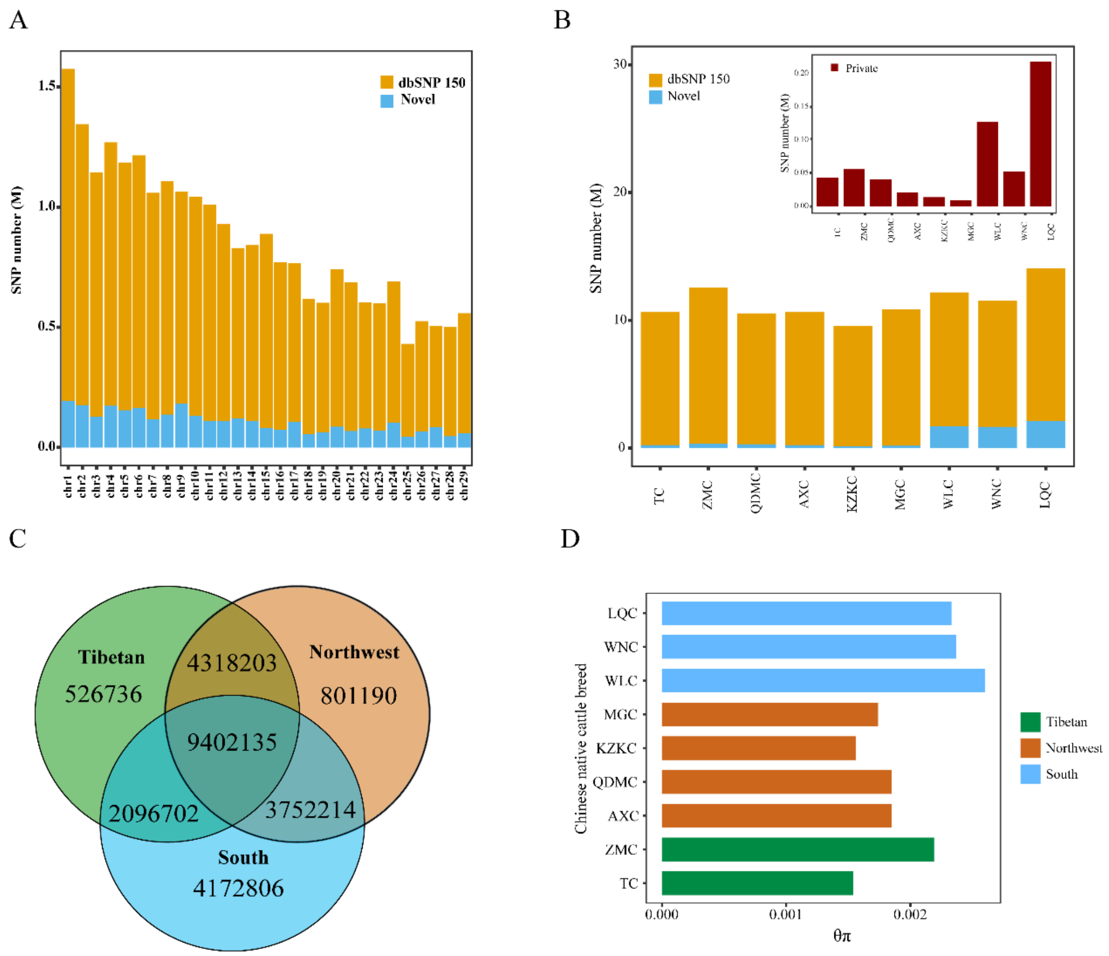

Figure S2. Summary statistics for genomic variation. (A) SNP distribution of 29 chromosomes in 27 individuals. (B) The number of single nucleotide polymorphisms per breed, the top right corner is the private SNP for each breed. (C) A Venn diagram of the shared SNPs among the three groups of Chinese native cattle breeds. (D) Average pairwise nucleotide distance (θπ) values of 9 native cattle breeds. The sky blue bars represent the number of novel SNPs, and the yellow bars represent the number of known SNPs (dbSNP150).

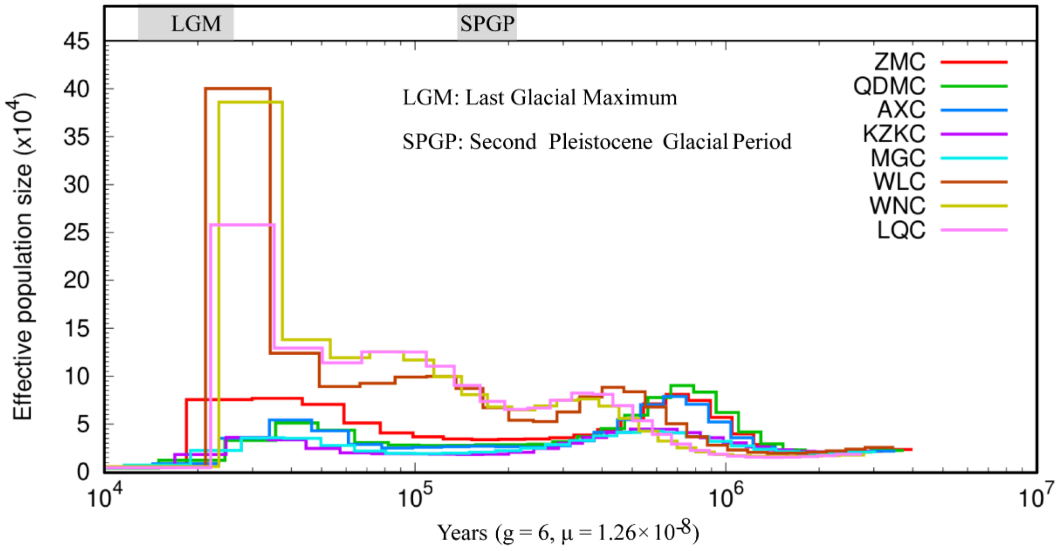

Figure S3. Pairwise sequential Markovian coalescent (PSMC) analysis results for the native cattle. The grey boxes represent the Last Glacial Maximum (LGM) and the Second Pleistocene Glacial Period (SPGP).

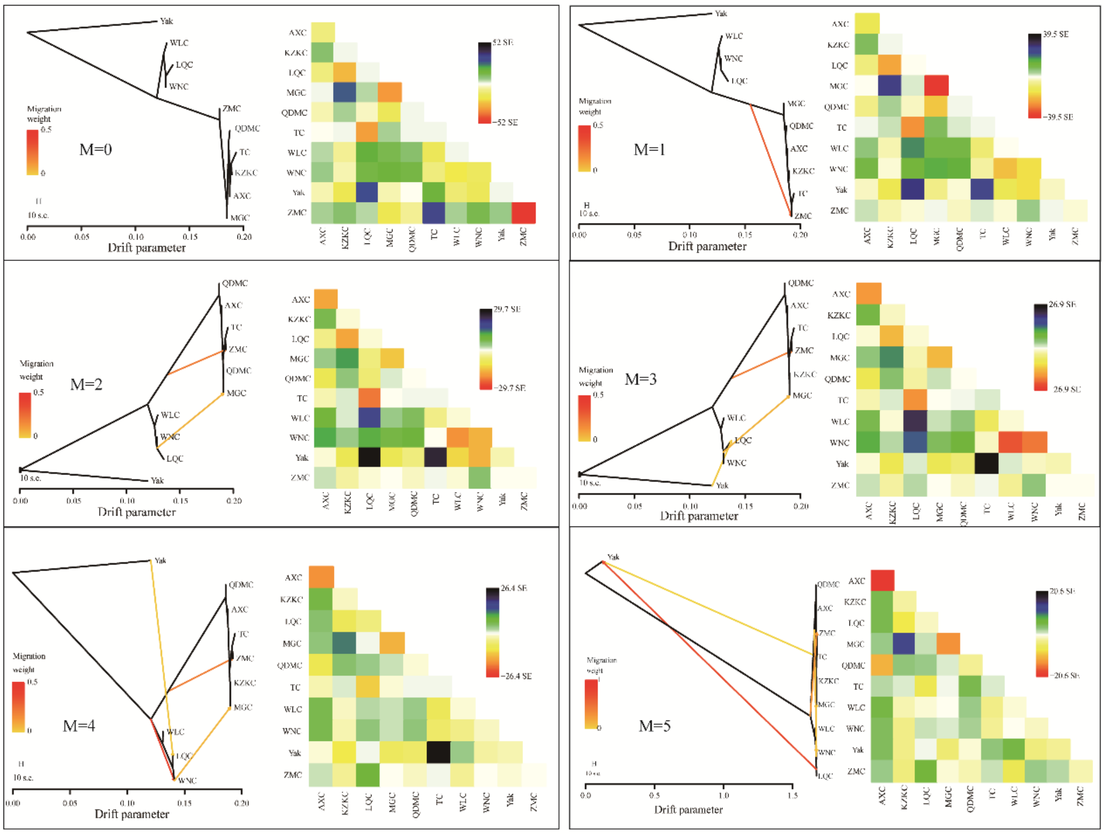

Figure S4. Genetic migration inferred by TreeMix (migration number from 1-5, 0 means no migration).

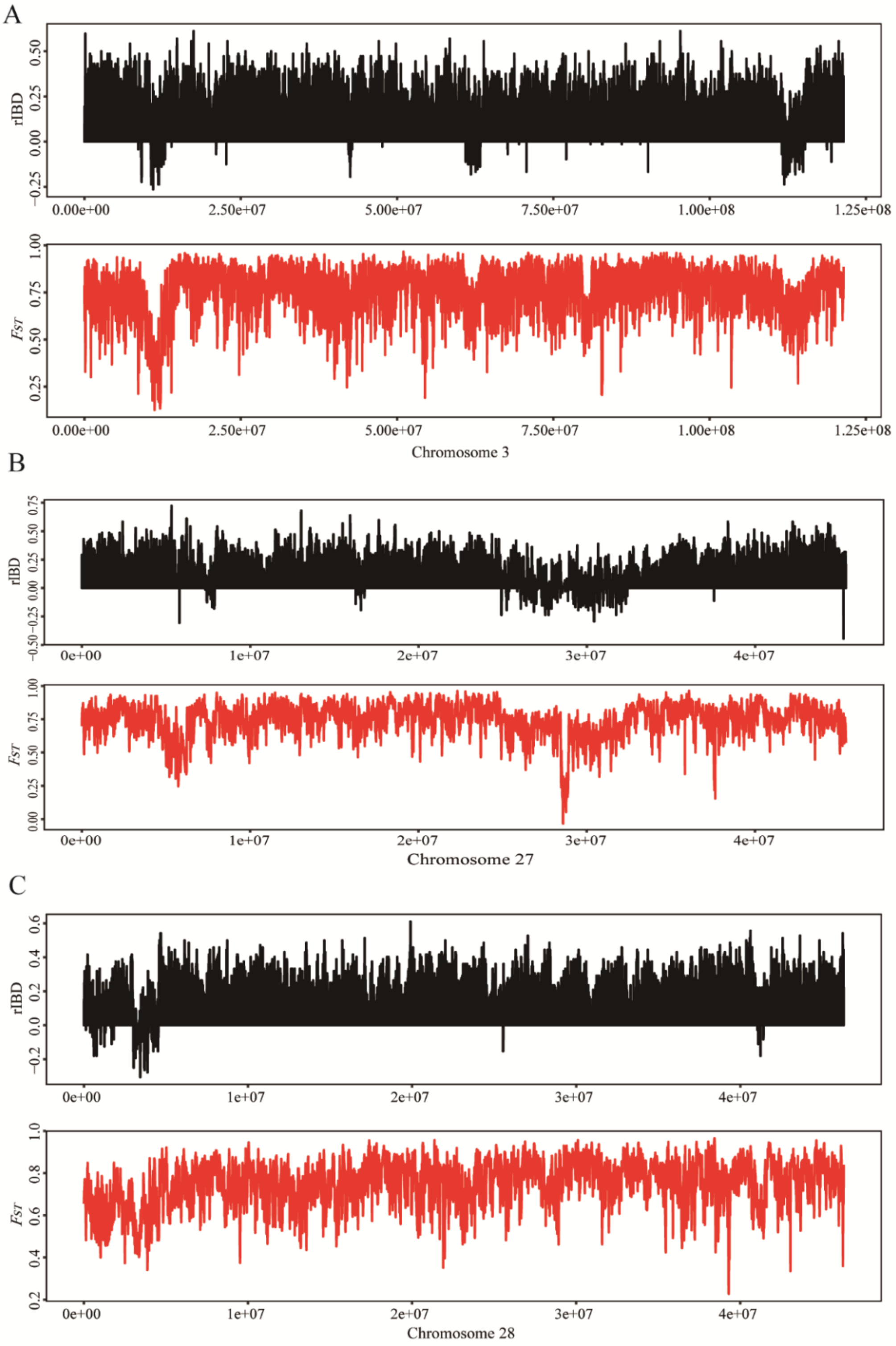

Figure S5. Values of rIBD and F_{ST} (between ZMC and Yak) of the three large regions. (A) chromosome 3, (B) chromosome 27 and (C) chromosome 28.

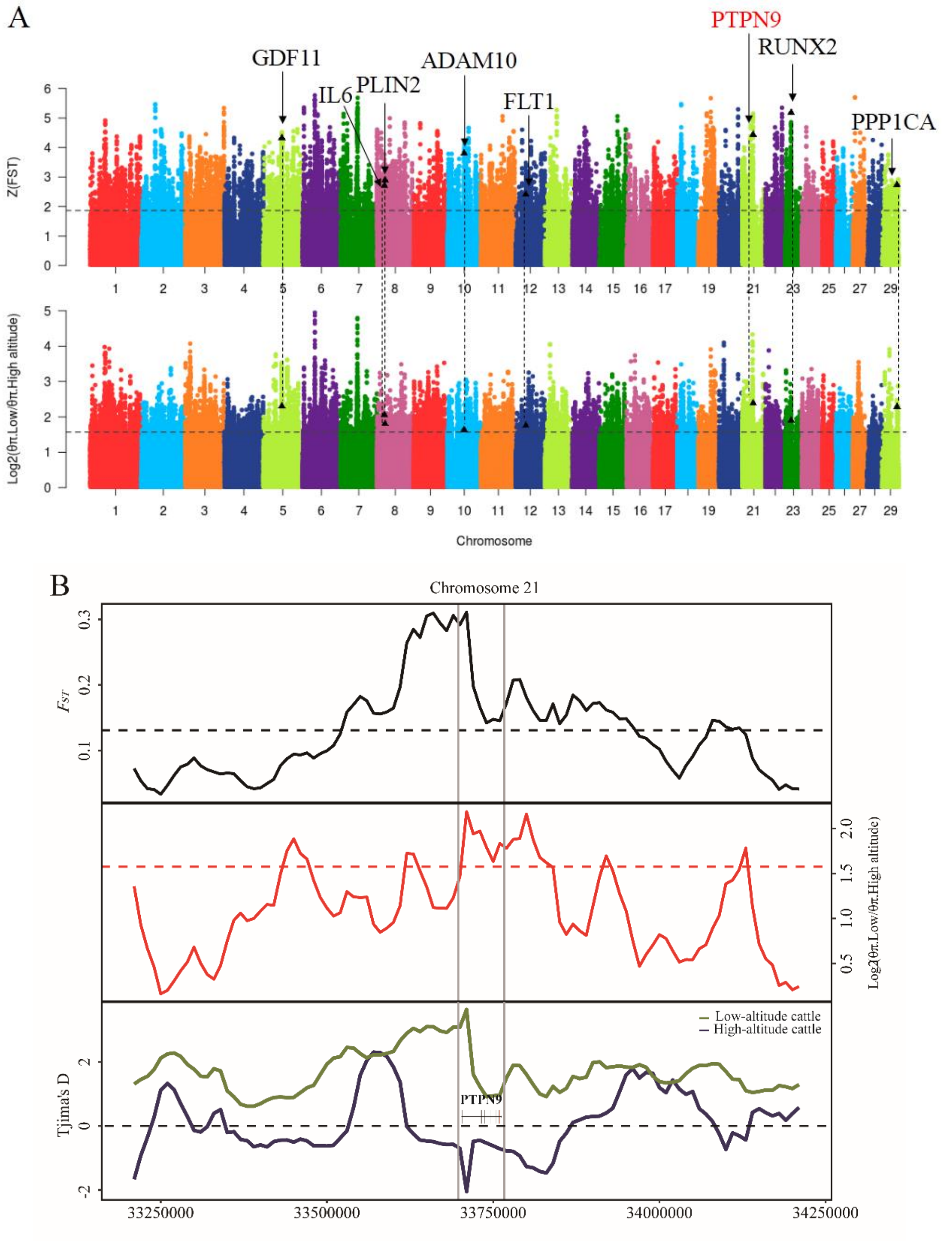

Figure S6. Genomic regions with strong selective sweeps in the high-altitude populations. (A) Distribution of log$_2$($\theta\pi$ ratio) and Z(F_{ST}) values calculated in 50-kb windows sliding in 10-kb steps. Windows above the dashed line (corresponding to the top 5% of the empirical log$_2$($\theta\pi$ ratio) distribution with values > 1.576 and the top 5% of the empirical Z(F_{ST}) distribution with values > 1.908) were identified as selected

regions for the high-altitude cattle. Strongly selected gene is marked in black triangle. $Z(F_{ST})=(F_{ST}$ $-\mu F_{ST})/\delta F_{ST}$, and θ_π ratios=θ_π-low-altitude/high-altitude (B) Example of genes with strong selective sweep signals in the high-altitude populations. $Log_2(\theta\pi$ ratio), F_{ST} and Tajima's D values are plotted using a 10-kb sliding window.

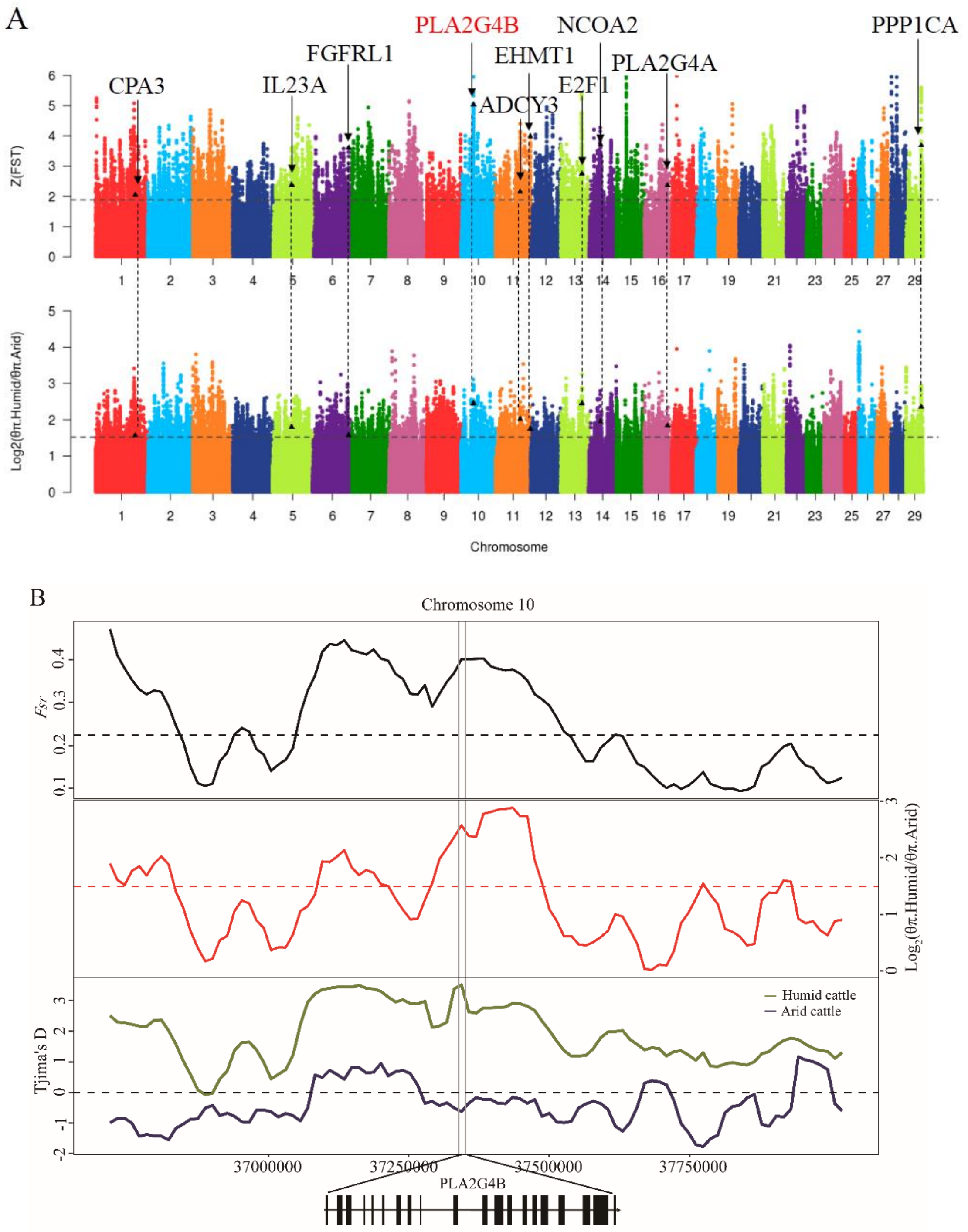

Figure S7. Genomic regions with strong selective sweeps in arid populations. (A) Distribution of $\log_2(\theta\pi$ ratio) and $Z(F_{ST})$ values calculated in 50-kb windows sliding in 10-kb steps. Windows above the dashed line (corresponding to the top 5% of the empirical $\log_2(\theta\pi$ ratio) distribution with values > 1.520 and the top 5% of the empirical $Z(F_{ST})$ distribution with values > 1.886) were identified as selected regions for the arid

population. Arid adaptation-related gene is labelled in black triangle. Z(F_{ST})=(F_{ST} - μF_{ST})/δF_{ST} and θ_π ratios=θ_π-humid/θ_π-arid (B) Example of gene with strong selective sweep signals in the arid population. Log_2($\theta\pi$ ratio), F_{ST} and Tajima's D values are plotted using a 10-kb sliding window.
